# An unusual case of necrotizing pneumonia in a healthy apnea diver

**DOI:** 10.1007/s15010-025-02617-6

**Published:** 2025-07-30

**Authors:** Christian Beyer, Rayya Alsalameh, Christian Bogdan, Marion Ganslmayer, Markus F. Neurath, Richard Strauß, Julia Fürst

**Affiliations:** 1 Medizinische Klinik 1, Gastroenterologie, Pneumologie und Endokrinologie, Universitätsklinikum Erlangen, Erlangen, Germany; 2 Mikrobiologisches Institut, Klinische Mikrobiologie, Immunologie und Hygiene, Universitätsklinikum Erlangen, Erlangen, Germany

**Keywords:** Hypervirulent Klebsiella pneumoniae, Pneumonia, Virulence factors, Difficult-to-treat

## Abstract

We report a case of necrotizing pneumonia caused by hypervirulent *Klebsiella pneumoniae* in an athletic, 54-year-old apnea diver. Since hypervirulent *Klebsiella pneumoniae* strains prevail in Southeast Asia and are mainly associated with “invasive liver abscess syndrome”, we considered and tested for hypervirulence only when the necrotizing pneumonia turned out to be refractory to initial antibiotic treatment. Although the hypervirulent *Klebsiella pneumoniae* strain in our patient did not show multi-drug resistance, a total of 6 months of high-dose antibiotic therapy was necessary to cure the infection.

## Introduction

Hypervirulent *Klebsiella (K.) pneumoniae* (hvKp) strains are an emerging threat for global health. While “classical” *K. pneumoniae* usually causes disease in immunocompromised and elderly individuals, hvKp can lead to severe and metastatic infections in healthy individuals [1–3]. HvKP exhibit specific virulence factors that support siderophore-mediated iron acquisition and capsule production and thereby help hvKP to evade host immune responses [4]. HvKp was first identified in Taiwan in 1986. Since then, it has mainly become prevalent in Southeast Asia. In recent years, it has been spreading globally with cases reported from North America and Europe [1–3]. While hvKP was originally described in the context of ‘invasive liver abscess syndrome’ [5], primary involvement of other organ systems is increasingly observed. Here, we report a case of community-acquired, necrotizing pneumonia with hvKP in an athletic, middle-aged apnea diver.

## Presentation of the case

A 54-year-old female high school teacher presented to our emergency department with shortness of breath, right-sided thoracic pain and fever. The patient had a history of allergic asthma and Hashimoto’s thyroiditis. She was taking hydrocortisone for adrenocortical insufficiency and she had suffered from rheumatoid arthritis in the past, from which she had recovered completely after treatment with TNF blockers. At presentation, the patient had not been taking any immunomodulatory agents for many years. Furthermore, the patient had a history of bilateral Achilles tendinitis caused by moxifloxacin, which had resolved after low-dose anti-inflammatory radiotherapy.

The patient was a semiprofessional apnea diver practicing and competing abroad. Six weeks prior to presentation, she had performed apnea dives in Egypt, but had returned healthy from this trip. The patient was married and did not have any children. Furthermore, the patient did not have any pets, but reported catching mice at her balcony to release them later in nature.

**Fig. 1:**
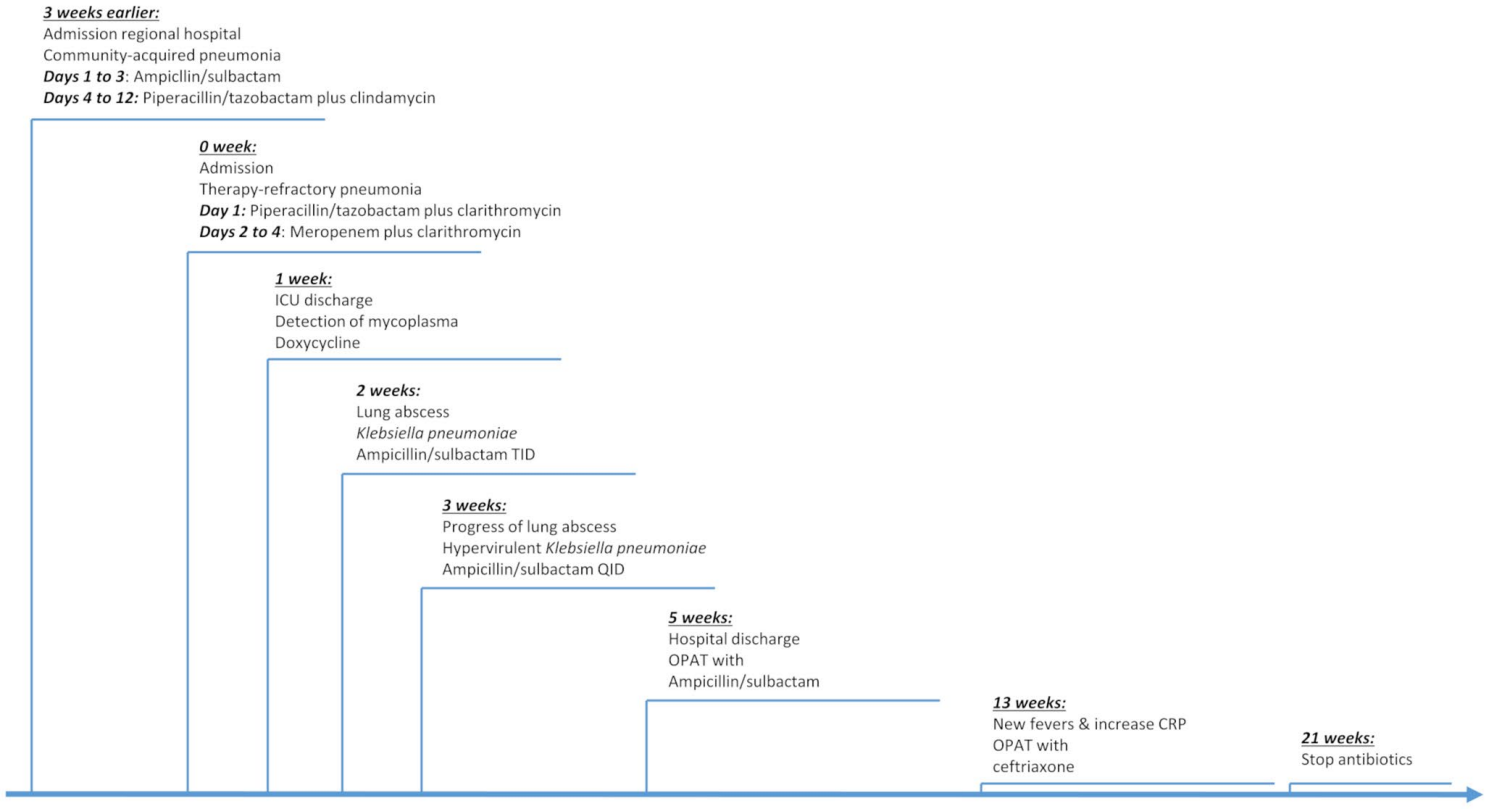
Time course indicating major events and antibiotic choices in the treatment of the patient with necrotizing pneumonia caused by hypervirulent *Klebsiella pneumoniae*

Three weeks prior to presenting at our hospital, the patient presented herself to a regional hospital with community-acquired pneumonia. Chest X-ray showed pneumonic infiltrates in the right upper lobe. The patient received parenteral ampicillin/sulbactam which was switched to piperacillin/tazobactam plus clindamycin due to insufficient clinical improvement after three days of treatment. Bronchoscopy with bronchoalveolar lavage (BAL) was scheduled, but later cancelled because the patient’s condition finally improved. The patient was discharged nine days prior to presentation at our emergency department (Fig. 1).

While being at home, symptoms were initially tolerable. Being equipped with a pulse oximeter for apnea practice, the patient recorded oxygen saturations between 88 and 90%. Immediately upon developing severe right-sided thoracic pain and fever, the patient went to our emergency department.

**Fig. 2:**
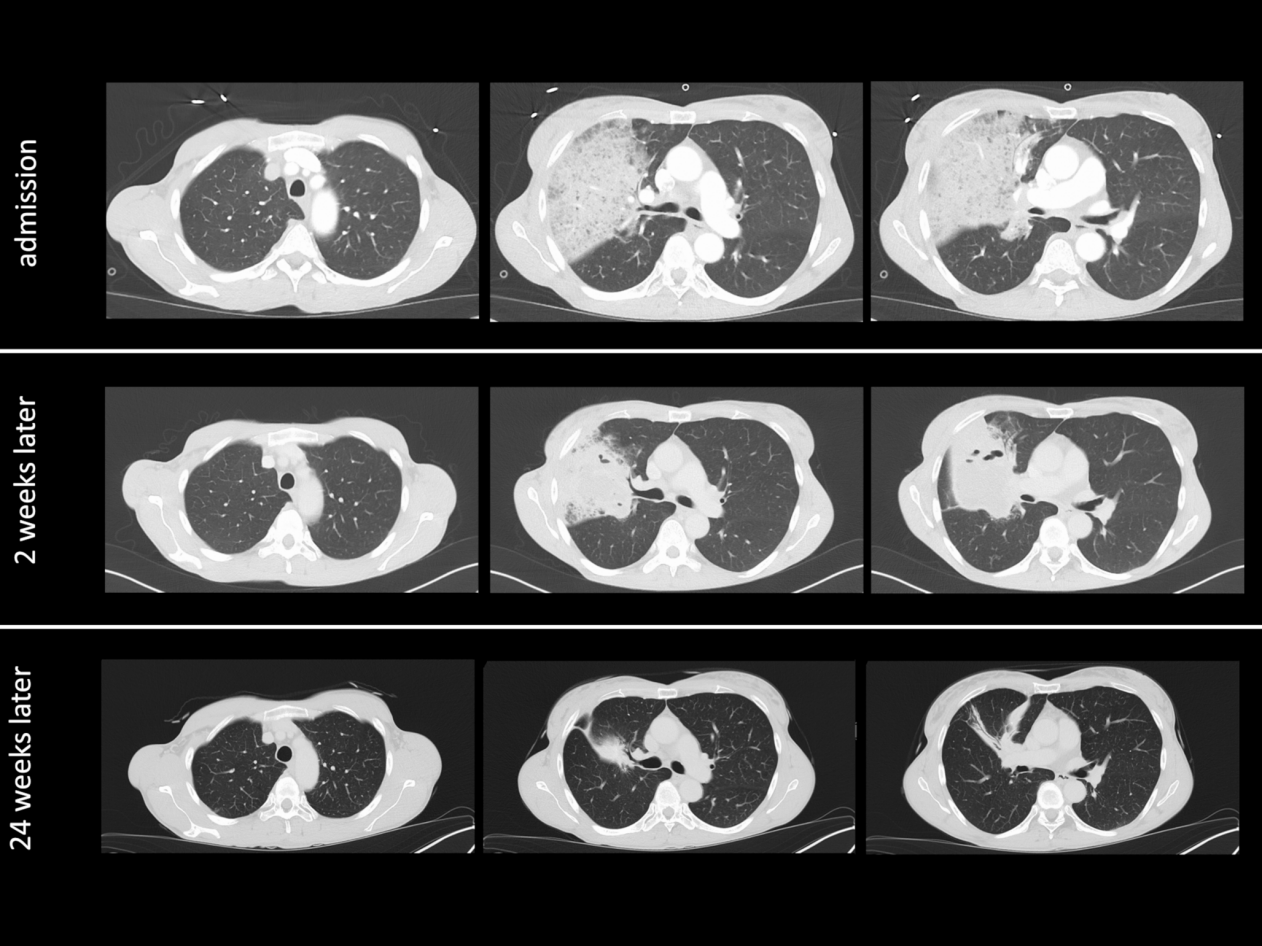
Chest CT scans in the lung window at various time points during the course of the necrotizing lung infection. Chest CT scans of three time points are shown: Time of admission, two weeks after admission when the lung abscess first became apparent, and at 24 weeks after admission when only residual changes were observed and antibiotic therapy was discontinued. For each time point three images are presented: At the levels of the aortic arch, the tracheal bifurcation and the bronchial segmentation

On admission, the respiratory rate was 31/min and the patient needed 5 L/min oxygen supplementation through a nasal cannula to obtain peripheral oxygen saturations above 90%. Leukocyte count was 22.000/µL. C-reactive protein was 13.7 mg/L but increased to 133.7 mg/L the following day. By then, procalcitonin was positive with 21.4 ng/mL. A CT scan demonstrated infiltrates in the right upper lobe (Fig. 2).

The patient received a diagnosis of “refractory pneumonia with pleuritis”. Treatment with piperacillin/tazobactam plus clarithromycin was initiated and the patient was transferred to our intensive care unit (ICU). Within the next day, oxygenation deteriorated and the patient received oxygen supply through high-flow nasal cannula. Treatment was switched to meropenem plus clarithromycin due to the travel history and the previous antibiotic treatment in the regional hospital.

Because of impending endotracheal intubation, bronchoscopy with BAL was postponed. In addition to blood cultures and *Legionella* urinary antigen testing, gargle fluid was collected to test for *Legionella*, *Chlamydia* and *Mycoplasma* infection by PCR. To our surprise, gargle fluid tested positive for *Mycoplasma (M.) pneumoniae*. Therefore, clarithromycin was changed to doxycycline.

The patient’s condition and inflammation markers improved rapidly within the next four days. Oxygen supply could be switched back to nasal cannula supplying 5 L oxygen/min. Meropenem was stopped after three days of treatment and doxycycline was continued. The patient was transferred to our general ward, where oxygen could be tapered off.

**Fig. 3:**
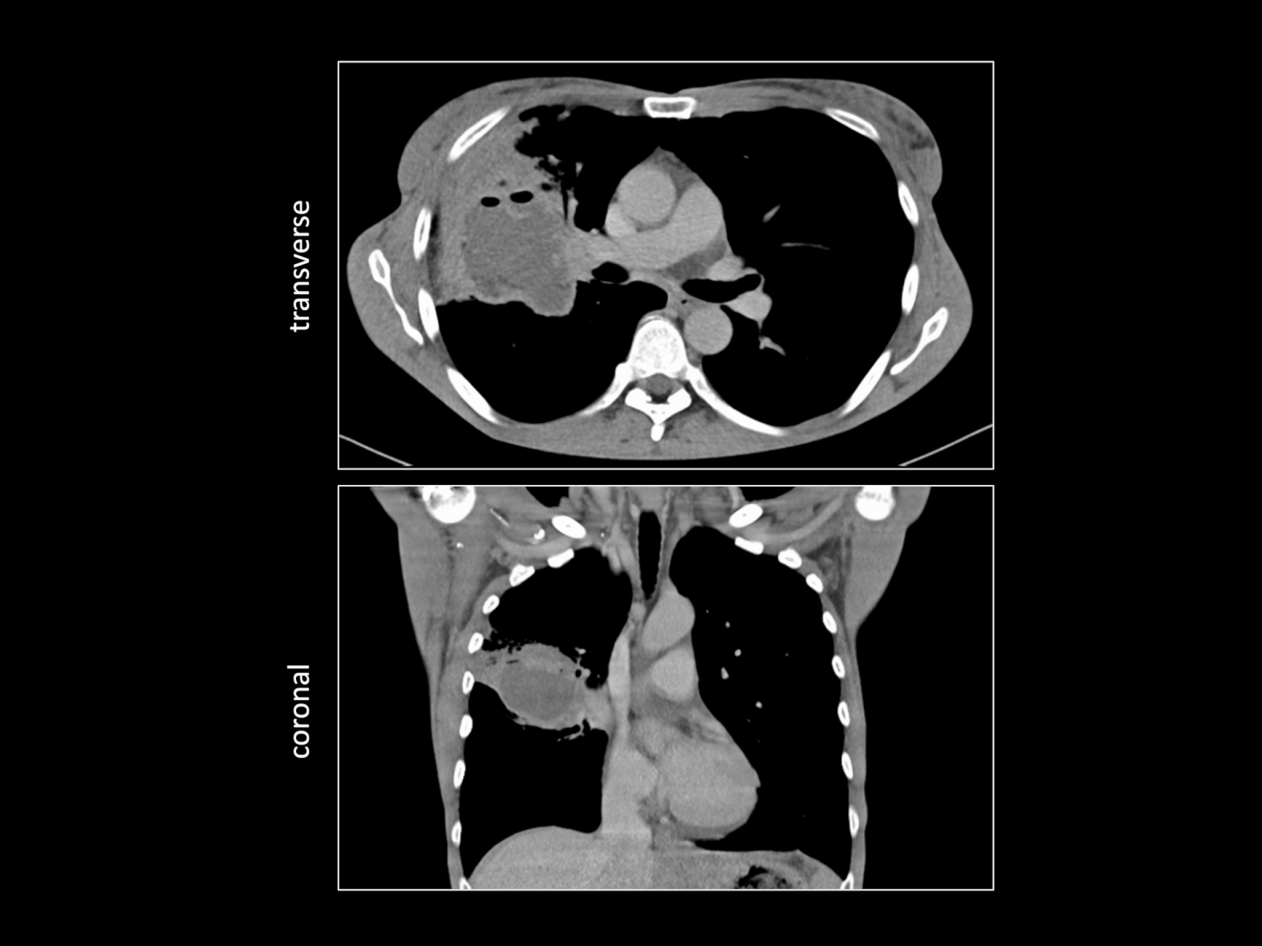
Chest CT scans in the soft tissue window demonstrating the lung abscess. CT imaging was done two weeks after admission, when lung abscess first became apparent. Two representative images in transverse and coronal plane are demonstrated

Ten days after discharge from the intensive care unit, the patient became febrile again and oxygen supplementation of 2 L/min through nasal cannula became necessary. Moreover, her inflammatory markers rose and CT scan showed a new lung abscess with an approximate size of 5 cm x 4 cm x 4 cm (Figs. 1 and 3) in the right upper lobe, where the infiltrates had resided. Now, bronchoscopy and BAL were performed and meropenem was restarted.

BAL revealed *K. pneumoniae* in large quantities and a positive aspergillus antigen-index of 2.4 (normal < 0.5). Retesting for *M. pneumoniae* was negative. Considering the patient’s history of apnea diving, PCR and culture for atypical mycobacteria were performed but remained negative. BAL cytology showed more than 90% of neutrophils. *K. pneumoniae* was resistant to ampicillin, but sensitive to other standard treatment options, including ampicillin/sulbactam. Therefore, treatment with meropenem was stopped and ampicillin/sulbactam was started. Although chest CT scan did not show signs of aspergillosis, isavuconazole treatment was initiated because of the highly elevated aspergillus antigen-index. Of note, differential blood count, lymphocytic differentiation, immunoglobulin levels and HIV serology were normal.

Since the lung abscess grew larger as demonstrated by follow up CT scan one week later, another bronchoscopy with BAL was performed. Again, *K. pneumoniae* was detected in large quantities. Both aspergillus antigen-index and aspergillus PCR remained negative. *Herpes simplex virus (HSV)-1* was now detected in moderate concentration. Because of the severity of necrotizing pneumonia despite adequate treatment in an apparently healthy individual, testing for specific virulence factors of hvKP was initiated (test platform: eazyplex^®^-hv-*K.pneumoniae*, AmplexDiagnostics GmbH, Germany). Indeed, PCR was positive for *aerobactin*, *regulator of mucoid phenotype A (rmpA)* and *rmpA2* genes, which was highly suggestive of hvKP.

While isavuconazole was stopped, ampicillin/sulbactam was continued with doses increased from 2 g/1 g TID to 2 g/1 g QID. The patient did not receive anti-viral treatment because *HSV-1* viral load was only moderately high and the CT was not suggestive of viral pneumonia. The following two weeks, the lung abscess regressed. After a total of five weeks of inpatient care, the patient was discharged on outpatient parenteral antibiotic therapy (OPAT) with ampicillin/sulbactam QID.

During outpatient treatment, the clinical improvement of the patient remained tedious. While the size of the lung abscess decreased slowly, the patient developed new bouts of fever, increased inflammation markers and new infiltrates in the middle and upper lobe after six weeks of OPAT. Another bronchoscopy with BAL detected *K. pneumoniae*, but no other pathogens. Therapy with ampicillin/sulbactam was continued.

After eight weeks of OPAT, the patient again demonstrated spikes of fever and an increase in inflammation markers. By then, antibiotic therapy was adjusted from ampicillin/sulbactam to ceftriaxone 2 g OD. After 12 weeks of OPAT, the lung abscess has finally resolved. Since chest CT scan still demonstrated infiltrates predominantly in the middle lobe and since inflammation markers were still elevated, OPAT was continued. Finally, after 16 weeks of OPAT and 21 weeks of total antibiotic treatment, chest CT scan only showed residual inflammatory changes within the lung parenchyma. Inflammation markers were normal. Eventually, antibiotic therapy was stopped.

Four weeks after discontinuing antibiotic treatment, the patient did not show any clinical signs of recurrent disease and inflammation markers remained normal. Eager to rebuild her apnea diving practice, our patient undertook lung function testing at that time: Vital lung capacity was almost back to baseline compared to her lung function prior to necrotizing pneumonia with hvKP.

## Discussion

Originally, hvKP was thought to be restricted to Southeast Asia, where its main clinical manifestation is “invasive liver abscess syndrome” [5]. While “classical” *K. pneumoniae* infections are mainly found in immunocompromised and elderly people, hvKP may also affect young and healthy individuals. The current disease model of “invasive liver abscess syndrome” proposes that hvKP strains colonizing the gut cause liver abscesses and metastatic infections in various organs [1, 4, 6], such as the eyes [7–9], meninges [10], and lungs [11]. Due to its metastatic character, hvKP infections may be confused with metastatic malignancies or other metastatic infections, such as tuberculosis [11].

Our case highlights the evolving epidemiology and pathogenicity of hvKP: Our patient did not report a history of travelling to Southeast Asia and she did not suffer from liver abscess, but she presented with lobar pneumonia before developing lung abscess, suggesting primary lung infection with hvKP. In line with our findings, reports from ICUs in France have shown that, apart from liver abscesses, hvKP causes community-acquired and aspiration pneumonias. In these settings, hvKP strains accounted for a significant portion of severe infections and were associated with high rates of multi-organ failure. The French study emphasized that many cases were independent from patients’ travel activities, suggesting a local acquisition and presence of hvKp within the French population [12]. A case series from a Chinese hospital in which several patients developed ventilator-associated pneumonias due to a carbapenem-resistent hvKP strain raises concerns for nosocomial infections through hvKP [13].

Reports describing primary infections by hvKP in organs other than the liver and lungs remain scarce. This may reflect specific pathophysiological aspects or underrecognition and underreporting, as awareness of hvKP outside Asia is still limited. Expression of specific virulence factors, pathogen-host-interactions, and environmental circumstances may all contribute to differences in the pathogenesis of “invasive liver abscess syndrome” compared to primary extrahepatic infections by hvKP [1, 3, 4, 7, 14], an research area, which still deserves further investigations.

Because of the history of apnea diving, we suspected ubiquitous mycobacteria (mycobacteria other than tuberculosis, MOTTs) as potential pathogens of treatment-refractory pneumonia. However, extensive microbiological studies excluded infection by ubiquitous mycobacteria. In this regard, the medical literature reports a relevant clinical role of ubiquitous mycobacteria for skin, but not for lung infections in divers [15].

The poor respiratory condition of our patient prevented early bronchoscopy and BAL. Besides blood cultures and urinary *Legionella* antigens, we used gargle fluid to screen for *Mycoplasma*, *Legionella* and *Chlamydia* spp. We were surprised by the positive result for *M. pneumoniae*, since CT studies were not suggestive of *Mycoplasma*. Given that the patient had already received five days of beta-lactam therapy, we opted to start doxycycline. In retrospect, however, *M. pneumoniae* probably did not play a major role in this case but initial beta-lactam treatment was too short to cure the infection by hvKP [16]. Results from the first BAL indicated a positive galactomannan-test suggesting infection with *Aspergillus* species. Again, clinical and radiographic findings were not typical for *Aspergillus* infection [17]. In a repeated bronchoalveolar lavage, galactomannan was normal, so that anti-fungal therapy was discontinued.

Upon detection of *K. pneumoniae* in the first BAL, we did not consider hvKP, since it is thought to be relatively rare in Western Europe and since the imaging studies did not show any signs of a liver abscess. Because antibiotic therapy did neither reverse necrosis nor bacterial load, we eventually suspected hvKP. Indeed, the *K. pneumoniae* isolate expressed the genes for aerobactin, regulator of mucoid phenotype A (rmpA) and rmpA2, indicating the presence of hvKP. Amongst many virulence genes associated with HvKP, genes regulating siderophore expression for efficient iron uptake and those regulating capsule production are considered hallmarks of hvKP. Phenotypic testing by using the so-called “string test” was not performed.

The medical literature reports that treating hvKP is often complicated by multi-drug resistance [1, 3]. Although sensitivity testing was favorable in our patient, initial beta-lactam and carbapenem treatment with relatively short treatment courses (a total of twelve days in the regional hospital and four days in our hospital) led to relapses. Finally, six months of antibiotic therapy was necessary to cure necrotizing pneumonia caused by hvKP. This highlights again, why hvKP is considered a global health threat.

## Conclusion

HvKP strains are an emerging global health threat. Although still rare in Western Europe and North America, necrotizing or metastatic disease affecting various organs and caused by *K. pneumoniae* should raise suspicion for hypervirulence. Various phenotypic or genetic approaches to test for hypervirulence are available, although a standardized approach still needs to be defined. Prolonged courses of parenteral and oral antibiotics are required to cure hvKP infections, especially with multi-drug resistance, which may complicate treatment regimens.

## Data Availability

No datasets were generated or analysed during the current study.
